# Milk Disposition Kinetics, Residue and Efficacy of Rifaximin After Intramammary Administration in Lactating Cow

**DOI:** 10.3390/antibiotics14121203

**Published:** 2025-12-01

**Authors:** Na Yu, Yaoxin Tang, Weifeng Zhao, Junhao Xiang, Jing Qu, Hao Wu, Yiming Liu

**Affiliations:** 1 National Feed Drug Reference Laboratories, Feed Research Institute, Chinese Academy of Agricultural Sciences, Beijing 100081, China; 821012420002@caas.cn (N.Y.); 821012410486@caas.cn (Y.T.); 15624345052@163.com (W.Z.); 82101235698@caas.cn (J.X.); 821012430496@caas.cn (J.Q.); 2 Key Laboratory of Animal Antimicrobial Resistance Surveillance, Ministry of Agriculture and Rural Affairs, Feed Research Institute, Chinese Academy of Agricultural Sciences, Beijing 100081, China; 3 Laboratory of Quality and Safety Risk Assessment for Products on Feed-Origin Risk Factor, Ministry of Agriculture and Rural Affairs, Feed Research Institute, Chinese Academy of Agricultural Sciences, Beijing 100081, China; 4 College of Veterinary Medicine, China Agricultural University, Beijing 100193, China

**Keywords:** rifaximin, milk disposition kinetics, residue, efficacy, cow milk

## Abstract

**Background:** Rifaximin is a non-aminoglycoside antibiotic utilized for the treatment of mastitis in cows, but its milk disposition kinetics, residue, and bacteriological status in lactating cow milk have hardly been reported. This study aimed to assess the milk disposition kinetics and residue of rifaximin in milk and to evaluate the bacteriological status in milk after intramammary treatment with rifaximin. **Methods:** An ultra-high-performance liquid chromatography–tandem mass spectrometry (UPLC–MS/MS) approach was developed to assess rifaximin concentrations in milk. Milk disposition kinetics parameters of rifaximin in cow milk were obtained by non-compartment model analysis. Rifaximin residues in milk were analyzed up to 108 h post-administration to estimate the withdrawal period. Clinically, the efficacy of Rifaximin Intramammary Infusion (Lactating Cow) was evaluated in mastitis cases caused by various pathogens and compared with lincomycin as the control drug, including clinical cure rate, bacteriological cure rate, and somatic cell count (SCC) at D21 post-treatment. **Results:** The C_max_ of rifaximin in milk was 54,273.3 ± 12,421.32 ng/mL, the area under the curve (AUC) was 340,731.8 ± 43,968.82 h⋅ng/mL, the T_1/2_ was 5.57 ± 0.68 h, the mean resident time (MRT) was 7.3927 ± 1.34 h, and the area under the moment curve (AUMC) was 2,475,745 ± 230,305.1 h⋅h⋅ng/mL. Based on rifaximin residues in milk, the withdrawal period for cow milk was calculated to be 95.1 h. Clinically, Rifaximin Intramammary Infusion (Lactating Cow) demonstrated a clinical cure rate of 83.33% and a bacteriological cure rate of 76.67% in mastitis cases caused by various pathogens, with both rates being 10% higher than those of lincomycin. At D21 post-treatment, the rifaximin group had a significantly lower SCC than the lincomycin group (*p* < 0.05). **Conclusions:** Rifaximin exhibits favorable milk disposition kinetics, an acceptable withdrawal period of 95.1 h, and good clinical and bacteriological cure rates in bovine mastitis. These findings support rifaximin as a useful intramammary option and contribute to rational antimicrobial use and milk safety in dairy.

## 1. Introduction

Mastitis is considered a significant disease, inflicting enormous economic damage to dairy farming, reducing production performance and increasing culling rates [1,2]. In addition, it also leads to detrimental impacts on the composition and quality of cow milk [3]. Mastitis in cows is primarily induced by pathogenic infection and can be categorized into clinical and subclinical forms predicated on the observable alterations in the mammary gland and milk [4]. *Streptococcus agalactiae* (*S. agalactiae*), *Staphylococcus aureus* (*S. aureus*), *Mycoplasma* spp., *Streptococcus dysgalactiae*, and *Corynebacterium bovis* are common pathogens that infiltrate the mammary glands of cows [5,6]. The therapeutic strategies for mastitis are primarily based on antibiotic therapy [7].

**Figure 1:**
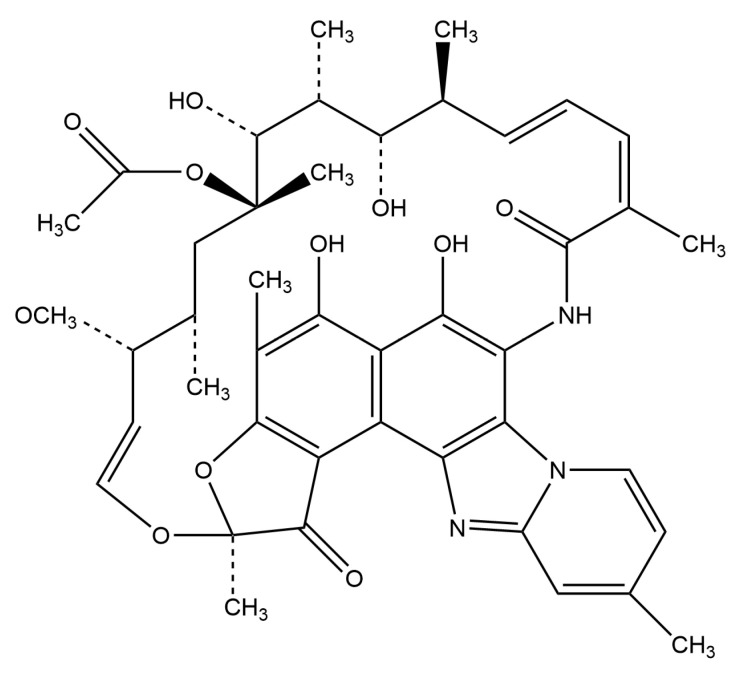
Molecular structure of Rifaximin.

Rifaximin, a semisynthetic antimicrobial, is derived from the parent compound rifamycin [8,9]. The molecular structure formula of rifaximin is shown in Figure 1. It has a high degree of antimicrobial activity against Gram-positive (including *Staphylococcus aureus*, *Staphylococcus epidermidis*, *Clostridium difficile*, etc.) and Gram-negative bacteria (including *Escherichia coli* (*E. coli*), *Salmonella*, *Shigella*, etc.) [9,10,11]. Rifaximin shares the same mechanism of inhibition as other rifamycin antimicrobials, binding to the DNA-dependent RNA polymerase subunit of bacteria, thereby inhibiting bacterial RNA synthesis [12]. The inclusion of an extra pyridoimidazole ring renders rifaximin nearly nonabsorbable [10,12,13]. Therefore, rifaximin is utilized for the therapy of gastrointestinal disorders based on this property. In the US, rifaximin has received approval for treating of adult irritable bowel syndrome with diarrhea (IBS-D), as well as for traveler’s diarrhea (TD) resulting from non-invasive *E. coli* strains [14,15]. In addition, rifaximin substantially diminishes the chances of hepatic encephalopathy (HE) recurrence in adults [16].

In the field of veterinary medicine, rifaximin has been used as a substitute for metronidazole for the management of persistent intestinal diseases in dogs [17], as well as in the treatment of mastitis and endometritis in dairy cattle [18,19]. Flammini et al. conducted the research on rifaximin on bovine endometrial progenitor cells and found that rifaximin exerted an anti-inflammatory effect [18]. Rifaximin is suitable for local treatment of endometritis, as it cannot penetrate epithelial cells, avoiding the withdrawal period prior to using milk or meat [20]. The application of rifaximin in cow mastitis has been approved by European drug evaluation agencies and was extensively utilized in numerous nations, including Italy, France, and Poland, South America, etc. [21]. Over the last several years, rifaximin has also been utilized to control and treat cow mastitis and several research trials have been performed [22,23,24]. Girardello et al. [25] studied the effectiveness of a new mastitis preparation (containing 100 mg rifaximin per syringe) in cows infected with Staphylococci or Streptococci on one or more quarters, investigating that 73 of 74 cows treated with rifaximin did not suffer from mastitis after calving. Of the 12 cows administered rifaximin at the onset of the dry period, none of them suffered from mastitis. Similar research by Avila et al. shows that rifaximin injected into the quarters during the dry period reduced the frequency of subclinical mastitis by 20% and decreased the occurrence of clinical mastitis [26]. Others demonstrated that rifaximin in combination with herbal preparations can prevent and treat cow mastitis in dry periods caused by *Staphylococcus aureus*, *Staphylococcus epidermidis* and *Streptococcus* spp. [27,28].

Pharmacokinetics underpins the determination of drug dosage and administration intervals, and assesses whether the drug can attain safe and effective concentrations at its site of action [29]. During the course of pioneering pharmaceutical development, pharmacokinetic studies are on an equal footing with pharmacodynamic studies and have proven a crucial component of preclinical and clinical research. Prior researchers have reported the pharmacokinetic characteristics of rifaximin in humans and animals following oral administration. Rifaximin was mostly present in feces and infrequently in urine, and it was nearly undetectable in blood [30,31]. Therefore, we conducted a milk disposition kinetics study of rifaximin intramammary infusion in cows to describe the kinetic characteristics of rifaximin in local target tissues (mammary gland/milk) of cows, which differs in purpose and interpretation from classical plasma concentration-based systemic pharmacokinetic studies.

In the process of animal husbandry, the abuse of veterinary drugs may lead to the accumulation of drugs in the animal’s body, resulting in contamination of animal-derived food and posing a risk to public health [32,33]. Due to the nearly nonabsorbable nature of rifaximin, this drug residue is likely to exacerbate the risk of excretion and deposition in the environment, exacerbating the existing threat of antibiotic resistance and the selection and transmission of various bacterial genes responsible for it. Therefore, it is imperative to create a reliable, rapid, sensitive, and simple method for detecting the rifaximin residues, which holds immense importance for the protection of people’s food safety and health. The most common method for the detection of rifaximin is the liquid chromatography (LC) method, but this method is mostly used for the detection of Active Pharmaceutical Ingredients (APIs) or preparations, while the detection of rifaximin in biological samples only is rare. High-performance liquid chromatography (HPLC) was considered for the British Pharmacopoeia and European Pharmacopoeia monograph on rifaximin in raw materials [34]. There are no official regulations guiding the detection of rifaximin in milk.

The present research’ s objective was to establish a UPLC-MS/MS detection method for rifaximin in milk in order to investigate its milk disposition kinetics of rifaximin, and to study the residue of rifaximin in milk after intramammary administration to determine its withdrawal period time. The therapeutic efficacy of rifaximin in dairy cows with clinical mastitis was investigated by evaluating the clinical cure and bacteriological cure. The studies of the milk disposition kinetics and efficacy of rifaximin could provide rational guidance to dairy farmers, and the determination of the milk withdrawal period of rifaximin can ensure the food safety of dairy products.

## 2. Results and Discussion

### 2.1. Optimization of Sample Extraction

The aim of sample pretreatment is to fully extract the main components while reducing the interference of excipients. In order to detect rifaximin residues in milk, protein and fat must be removed from milk samples. Antibiotics such as cephalosporins, tetracyclines, macrolides, fluoroquinolones and sulfonamides are usually extracted from milk using acetonitrile [35,36,37,38], and then purified by SPE after extraction. We compared the extraction effect of acetonitrile and methanol and the recoveries of rifaximin were 98% and 92% for acetonitrile and methanol, respectively. Therefore, acetonitrile was finally chosen as the extraction solution.

### 2.2. Selection and Optimization of Chromatographic Conditions

**Figure 2:**
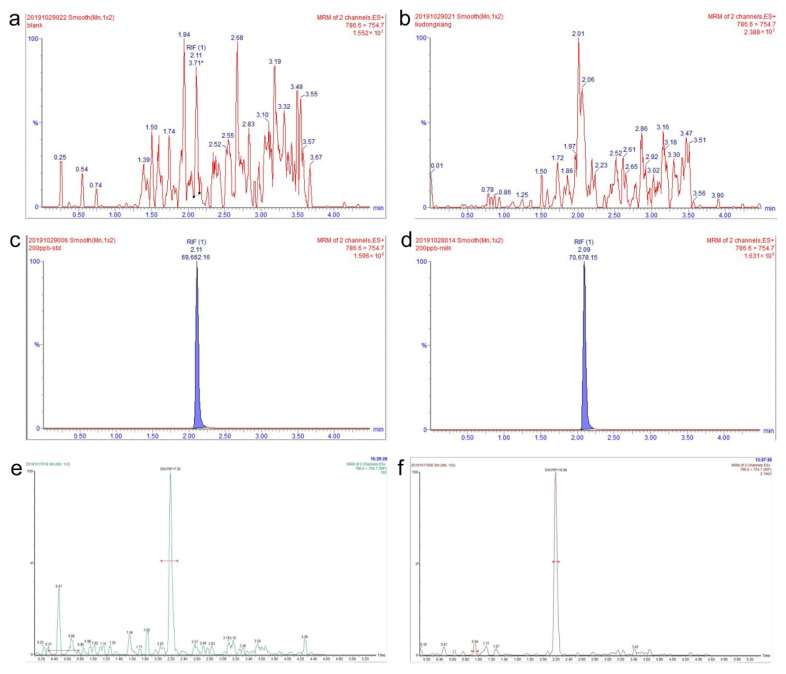
UPLC-MS/MS chromatograms. (**a**) of blank mobile phase, (**b**) of blank milk, (**c**) of blank mobile phase spiked with rifaximin (200 μg/kg), (**d**) of blank milk sample spiked with rifaximin (200 μg/kg), (**e**) for LOD of rifaximin in blank milk, (**f**) for LOQ of rifaximin in blank milk. Note: * representing manual integration of the peak.

The separation of UPLC is greatly improved compared to conventional HPLC, which not only facilitates the ionization of compounds, but also reduces matrix effects to a certain extent, resulting in improved sensitivity and reproducibility [39]. The selection of the chromatographic column is contingent upon the molecular weight, solubility and other characteristics of the compound to be measured [40]. An ACQUITY BEH C18 column (2.1 mm × 100 mm, 1.7 μm) was selected for the detection of rifaximin. Figure 2 demonstrates that the column could separate rifaximin in a short time with good peak shape.

Adding 0.1% formic acid to the water could permit the high signal intensity, enhance mass response profile and improved sample ionization efficiency [41]. In addition to that, formic acid could provide H^+^ for the ESI+ mode. Therefore, formic acid in water/acetonitrile was used as mobile phase in this trial and the results met the requirements of the EU regulations, Commission Decision 2002/657/EC. A gradient elution was used, and the initial gradient condition was set to 30% B and increased linearly to 80% B within 3 min. For 3 to 4.5 min, the gradient condition was set to the initial condition and decreased linearly to 30%B. This condition achieved good separation effect and avoided the interference of other heterogeneous peaks. After optimizing the chromatographic conditions, the retention time of rifaximin was 2.1 min with good peak shape. Under these conditions, a sample was analyzed in 4.5 min, which saves analysis time and increases productivity.

### 2.3. Optimization of Mass Spectrometry Conditions

Rifaximin standard was dissolved in pure acetonitrile and configured into a standard solution of 100 mg/kg, which was injected into the ion source by direct injection with a syringe pump. Rifaximin was scanned in full scan mode. Based on the [M+H] abundance of drug molecules gathered in the ESI+ ion mode, the ion (*m*/*z* 786.6) was determined to be the precursor ion. Using the optimized precursor ion’s mass spectrum characteristics, the product ion was located using the secondary scanning method. We found two major fragment ions (*m*/*z* 754.7 and *m*/*z* 150.8), and the most abundant fragment ion (*m*/*z* 754.7) was chosen to be the quantitative ion. The other was treated as the qualitative ion.

### 2.4. Method Validation

#### 2.4.1. Selectivity and Matrix Effect

While utilizing ESI, the presence of matrix components influences the ionization of analytes [42]. In the current research, the peak area of rifaximin added to blank milk samples and that of rifaximin at an equivalent concentration in the mobile phase were compared in order to assess ME. By comparing Figure 2c,d, an ME value of 1.46% was calculated, indicating that the ME of rifaximin detected in milk samples was compliant. Comparison of Figure 2a–d demonstrated that no interferences corresponding to the retention time of rifaximin were observed in the blank mobile phase and blank milk. Thus, the method was highly specific.

#### 2.4.2. Linearity

The linear regression analysis of rifaximin was carried out using the least weighted squares method. The calibration curve of rifaximin was linear in the matrix-matched range of 1 ng/mL to 200 ng/mL, with a linear equation of Y = 351.122X + 282.03 and r > 0.999.

#### 2.4.3. LOD and LOQ

**Figure 3:**
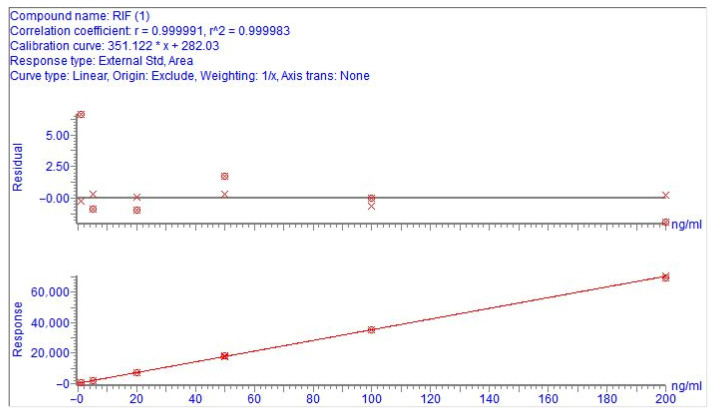
The calibration curves for rifaximin in milk.

The standardized working curves for the quantification of rifaximin in cow’s milk are shown in Figure 3. Rifaximin standards diluted with blank milk matrix were assayed, and the LOD was 0.5 ng/mL to satisfy S/N ≥ 3, and the LOQ was 1 ng/mL to satisfy S/N ≥ 10. Figure 2e,f show the results of the chromatograms for the LOD and LOQ of rifaximin in milk samples. The LOD of rifaximin in milk was 30 ng/mL and the LOQ was 150 ng/mL in the HPLC method established by Manjarrez et al. [20] Our method is more sensitive and can detect lower concentrations of rifaximin residues compared to the method of Manjarrez et al.

#### 2.4.4. Precision and Accuracy

The mean recoveries, inter-day coefficients of variation (inter CV) and intra-day coefficients of variation (intra CV) of rifaximin spiked in milk samples were given in Table S1. The mean recoveries of rifaximin spiked in milk were between 77.65% and 85.98%, and the intra CV were all within 12.78%, and the inter CV were all within 8.20%. The findings showed that the methodology we established is accurate, precise and it may be utilized for determining rifaximin in milk.

#### 2.4.5. Stability

The results of stability are given in Table S2. Rifaximin in milk was stable after being stored at 25 °C for 24 h or at −20 °C for 30 days and repeated freeze–thawing for three such cycles. The CV at each level was less than 7.0% and corresponds with acceptance criteria.

### 2.5. Milk Disposition Kinetics Results

**Figure 4:**
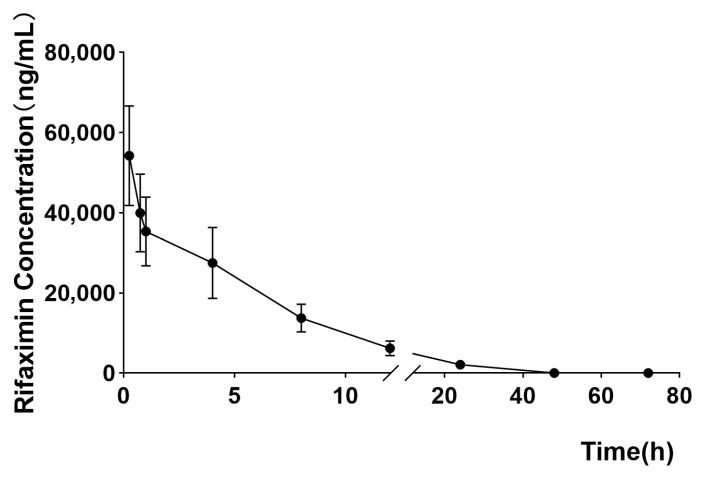
The curve of rifaximin concentration with time after single gland perfusion administration of 100 mg rifaximin gland injection once time (*n* = 12).

For intramammary infusion preparations, the concentration time curve of drugs in milk is the most relevant indicator for evaluating their local efficacy in treating bovine mastitis and determining the withdrawal period [43,44]. The mean milk concentration-time profiles of rifaximin, following an intramammary infusion of 100 mg/gland, are shown in Figure 4. Table 1 shows the primary milk kinetics parameters of the analytes. The milk kinetics profile of rifaximin in milk after intramammary infusion demonstrated a rapid decline in milk concentrations, with levels decreasing to 2086.7 ± 762.4 ng/mL at 24 h and approaching the 60 ng/mL by 48 h post-administration. Notably, milk concentrations fell below 10 ng/mL by 72 h, indicating effective clearance from the mammary gland. These findings suggest a favorable residue profile for rifaximin, supporting its use as an intramammary treatment in dairy cows while ensuring compliance with food safety regulations.

The peak concentration (C_max_) was 54,273.3 ± 12,421.32 ng/mL. The concentration of rifaximin in milk remained higher than the MIC_90_ of *Escherichia coli* (8 μg/mL), *Staphylococcus aureus* (0.125 μg/mL), and *Streptococcus agalactiae* (8 μg/mL) until 48 h. The AUC of rifaximin in milk was 340,731.8 ± 43,968.82 h⋅ng/mL, which represents the overall exposure of the drug in intramammary milk and is related to the duration of its efficacy. In contrast, the area under first moment curve (AUMC) was 2,475,745 ± 230,305.1 h⋅h⋅ng/mL, and the MRT of rifaximin was 7.39 ± 1.34 h. T_1/2_ is 5.57 ± 0.68 h, as the key parameter for determining the rate of rifaximin clearance from milk, which represents the speed at which rifampicin is eliminated in milk. Manjarrez et al. [20] reported lower AUC values (8650 h⋅ng/mL and 7750 h⋅ng/mL) and prolonged T_1/2_ values (23.9 and 24.6 h) following a similar 100 mg dose. The discrepancies may stem from differences in formulation and experimental design, particularly the larger sample size (12 cows) utilized in our study, which reduced the influence of individual variability. Furthermore, our optimized UPLC-MS/MS analytical method with a lower LOD (0.5 ng/mL) and LOQ (1.0 ng/mL), combined with higher recovery rates (77.65–85.98%), provides a more robust and sensitive assessment compared to the methodology employed by Manjarrez et al. The MRT of rifaximin (7.39 ± 1.34 h) and T_max_ (0.25 h) observed in our study highlight its swift distribution and elimination from milk, aligning with its limited systemic absorption previously reported in non-lactating models [12,30]. The virtual absence of systemic absorption is a key pharmacological advantage, minimizing concerns regarding systemic exposure and off-target effects. Such properties enhance the suitability of rifaximin for localized mastitis therapy, as it ensures high intramammary drug concentrations while maintaining low systemic residues.

Overall, our findings provide critical insights into the milk disposition kinetics of rifaximin following intramammary infusion and highlight its potential as an effective and safe treatment for bovine mastitis. The high intramammary drug concentrations, coupled with its rapid clearance and minimal systemic absorption, position rifaximin as a valuable therapeutic option for clinical practice. Further studies addressing the optimization of dosing regimens and resistance mitigation strategies are warranted to ensure the rational and sustainable use of rifaximin in lactating dairy cows.

### 2.6. Residue Results

Ensuring the absence of harmful antibiotic residues in milk is paramount to consumer safety. Milk, one of the most basic foods, provides necessary energy and nutrients for people of all ages [45]. Given the increasing global demand for milk, particularly in countries like China, where per capita milk consumption has significantly risen from 18 to 38 kg between 2007 and 2020 [46], ensuring the safety of milk consumed by humans is of paramount importance. Numerous harmful consequences, including allergies, mutagenicity, the introduction of microorganisms resistant to antibiotics into the body, carcinogenicity, reproductive problems, hepatotoxicity, and even the possibility of anaphylactic shock in humans, may be caused by antibiotic residues [32]. To this end, the EU has set up a complete legislative framework which stipulates the maximum allowable limit of veterinary medicines in food, so as to ensure people’s food safety. On the basis of the European commission decision (EU) No 37/2010 on pharmacologically active substances in food of animal origin and their classification, the MRL for rifaximin in milk is 60 μg/kg.

**Figure 5:**
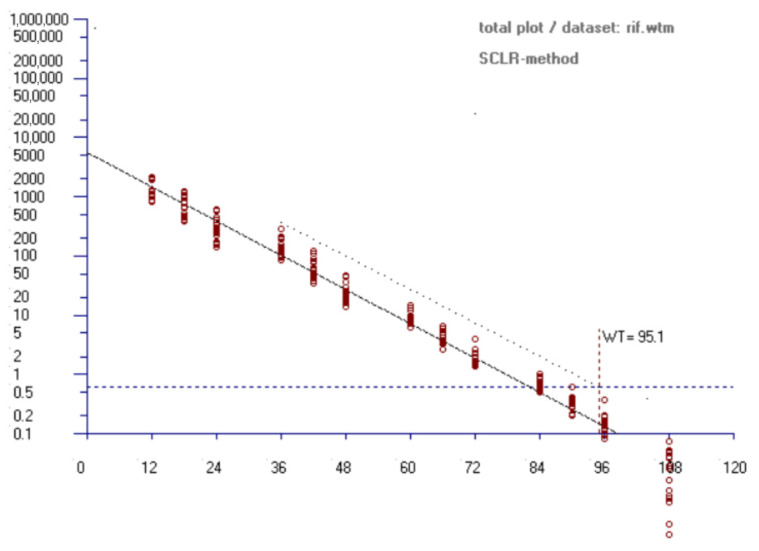
Estimated withdrawal times for rifaximin in cow milk (*n* = 20).

Using the validated UPLC-MS/MS method, we monitored the depletion of rifaximin residues in milk following intramammary administration. The average concentration of rifaximin in milk at 12 h, 18 h, 24 h, 36 h, 42 h, 48 h, 60 h, 66 h, 72 h, 84 h, 90 h, 96 h and 108 h post-administration are shown in Table 2. At 90 h after the last dose, the mean concentration of rifaximin in milk was 32.86 ± 9.90 μg/kg, which is below the MRL for rifaximin in milk specified by the EU, which is 60 μg/kg. The withdrawal period for rifaximin intramammary infusion in milk was determined to be 95.1 h (approximately 4 days) using WTM1.4 software (Figure 5). The results presented in this study are critical for defining practical treatment guidelines, as an accurate withdrawal period can reduce economic losses for farmers by determining the interval during which milk must be discarded.

Guaranteeing that milk is free from detectable levels of harmful antibiotic residues is crucial for consumer safety. Although rifaximin is not absorbed orally [30,31], and residues of rifaximin in food of animal origin and its deleterious consequences on people have not been reported, establishing a compliant milk withdrawal period (WP) remains a regulatory necessity to guarantee that residue levels do not exceed the maximum residue limit (MRL) of 60 μg/kg established by the EU (Commission Regulation (EU) No 37/2010).

It is worth noting that while our study provides compelling evidence for a shorter withdrawal period, additional research is required to comprehensively assess the potential effects of rifaximin intramammary infusion residue on animal health, milk quality, and consumer safety.

### 2.7. Clinical Cure

**Figure 6:**
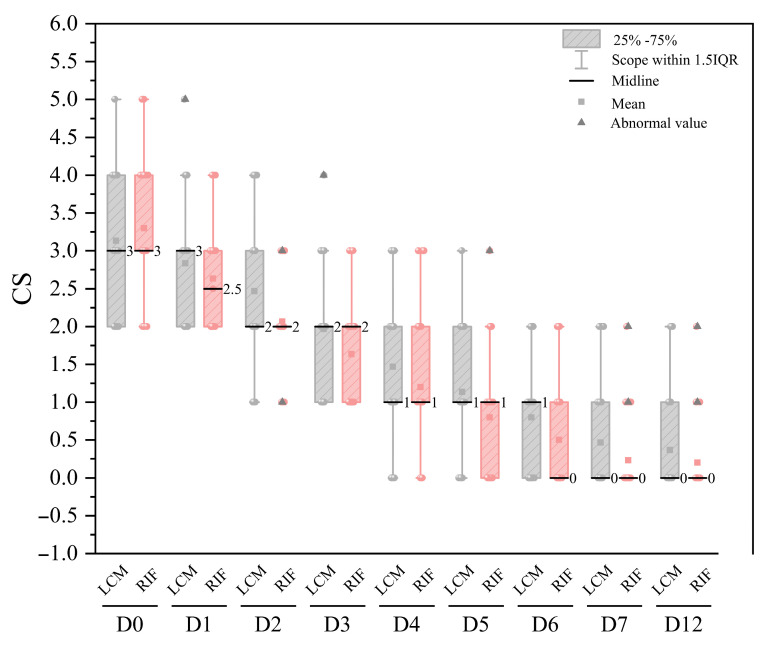
The results of clinical scores for rifaximin group and lincomycin group (*n* = 60).

Clinical outcomes were widely used by farmers and researchers as an intuitive indication of the success of intramammary therapy [47,48]. In this research, cows with a score of 0 were categorized clinically cured. The Shapiro–Wilk test confirmed that the clinical score data did not follow a normal distribution at any time point (*p* < 0.05). Therefore, all clinical score data are now presented as median (interquartile range, IQR) as given in Figure 6. Regarding the median CS at each observation point in the rifaximin group, a significant treatment effect was observed from D1 to D12 compared to the pre-administration(D0) (*p* < 0.001) used the Wilcoxon signed-rank test. However, there was no significant difference (*p* > 0.05) in clinical scores between D7 and D12, suggesting that most cows had recovered by D7. The lincomycin group also showed the same trend as the rifaximin group, with no significant difference (*p* < 0.05) in clinical scores on D7 and D12. For comparisons between the rifaximin and lincomycin groups at each time point, the Mann–Whitney U test was employed. No statistically significant differences in clinical scores were found between the two groups at any time point other than D2 (*p* < 0.05). The results of clinical cure rates in each group are shown in Table 3. Statistical analysis revealed a significant superiority in the clinical cure rate of the Rifaximin group over the Lincomycin group at D5 (43.33% vs. 20.00%, χ^2^ = 3.913, *p* = 0.048) and D6 (66.67% vs. 43.33%, χ^2^ = 3.333, *p* = 0.048), supporting a more rapid treatment effect for Rifaximin in the mid-term. The shortest cure days of rifaximin intramammary infusion in the experimental group and lincomycin intramammary infusion (lactating cow) in the control group was 4 days. Rifaximin intramammary infusion showed good efficacy against clinical cow mastitis, with a cure rate of up to 83.33% and an average number of days to cure of 5.64 days, compared with the control drug lincomycin, which showed a cure rate of 73.33% and an average number of days to cure of 6.41 days. Although the superior clinical performance of rifaximin was not statistically significant, it generates the hypothesis that its known dual antibacterial and anti-inflammatory properties [49] might contribute to a therapeutic benefit. This potential link is consistent with the positive bacteriological and SCC outcomes observed in this study but requires confirmation through future research with appropriately powered sample sizes.

Future studies ought to concentrate on the long-term impacts of rifaximin in treatment of mastitis, including its effects on recurrence rate, milk yield recovery, microbial resistance patterns, environmental safety, and economic feasibility. Extensive field trials are necessary to confirm the efficacy and safety of rifaximin in commercial farming environments. By addressing these questions, the use of rifaximin can be optimized to enhance udder health, improve milk quality, and meet the demands of modern dairy production systems.

### 2.8. Bacteriological Cure

Our findings demonstrate that rifaximin was more effective than lincomycin in reducing the bacterial load in milk. At D21 post-treatment, the pathogen detection rate in the rifaximin group was significantly lower (23.33%) compared to the lincomycin group (33.33%) (*p* < 0.05), indicating a more favorable bacteriological cure rate. The treatment protocols used in this study followed established guidelines, including multiple sampling points for bacteriological analysis (D −1, D0, D14, and D21), which allowed for a more accurate assessment of the treatment’s efficacy over time [43]. In this experiment, we sampled twice before treatment (D −1 and D0) and after treatment (D14 and D21) for bacteriological cultures, respectively. Bacteriological culture results are presented in Table 4. The pathogens separated from milk samples were *S. aureus*, *S. agalactiae*, and *E. coli*. The number of quarters infected with *S. aureus* was relatively uniformly distributed across treatments, with four quarters detected in rifaximin treatment group and five quarters detected in lincomycin treatment group. In the quarters of dairy cows with clinical mastitis, 28 quarters in the rifaximin group had only a single bacterial species cultured, and no mixed cultures were found, while 26 quarters in the lincomycin group had the same results. The rifaximin group identified one quarter infected with both *E. coli* and *S. agalactiae* and one cow infected with *E. coli* and *S. aureus*. The lincomycin group identified two quarters simultaneously infected with *E. coli* and *S. agalactiae*, and two quarters infected with *E. coli* and *S. aureus*.

For the Gram-negative pathogen *E. coli*, the bacteriological detection rate in the rifaximin group was 83.33% at D0 and 6.67% at D21. In contrast, the bacteriological detection rate in the lincomycin group was 90% at D0 and 16.67% at D21. This finding is consistent with the good bacteriostatic effect of rifaximin on *E. coli* reported in previous research [11,50]. The cure rate of *E. coli* in the rifaximin group was numerically higher than that in the lincomycin group (92.0% vs. 81.5%), but the difference did not reach statistical significance (*p* > 0.05). Due to the limited sample size of this study, this finding needs to be further validated in larger clinical trials. The cure rate of Escherichia coli in the rifaximin group was numerically higher than that in the lincomycin group (92.0% vs. 81.5%), but the difference did not reach statistical significance (*p* = 0.428). Due to the limited sample size of this study, this finding needs to be further validated in larger clinical trials. The superior cure rate for *E. coli* in the rifaximin group supports its potential as an effective treatment for clinical mastitis caused by this pathogen. For the Gram-positive pathogen, the bacteriological cure rate for *S. aureus* was 25% (one cured/four infected) in the rifaximin treatment group, which is notable given the well-documented challenges associated with eradicating this pathogen in mastitis treatment. *S. aureus* is recognized for its capacity to produce biofilms, which contribute to its persistence in the mammary gland and resistance to antibiotic treatment [51]. The incomplete eradication of *S. aureus* in this study could be attributed to the pathogen’s intrinsic mechanisms of immune evasion and detoxification [52], which often reduce the efficacy of many antibiotics, including rifaximin. Although the cure rate of *S. aureus* in the rifaximin group (25%) was higher than that in the lincomycin group (0%), this difference was not statistically significant (*p* > 0.05, Fisher’s Exact Test). After treatment with rifaximin, the detection rate of *S. agalactiae* decreased from 10% at D0 to 6.67% at D21. In contrast, the detection rate of *S. agalactiae* remained unchanged at 6.67% at both D0 and D21 in the lincomycin group. The in vitro antimicrobial susceptibility of the predominant mastitis pathogens isolated from the study farm was evaluated to inform the selection of isolates for the subsequent in vivo efficacy trial and to provide context for the clinical outcomes. Due to the single-farm origin of all isolates, which implies potential clonality, the detailed MIC distributions for *E. coli*, *S. aureus*, and *S. agalactiae* are provided as Supplementary Material (Table S3). Notably, the observed MIC values for all three pathogens were generally low, indicating no evidence of resistance development in the circulating herd strains against the tested drug. This trend of high in vitro susceptibility is consistent with the promising bacteriological cure rate of 76.67% observed in the clinical trial conducted on the same farm. However, the disparity between the excellent MIC profile of *S. aureus* and the modest in vivo cure rate may be attributed to the biofilm-forming nature of *S. aureus* [53], which can protect bacteria from antibiotics regardless of in vitro susceptibility, highlighting the challenges of treating biofilm-associated infections.

### 2.9. Results of Milk Somatic Cell Counts

**Figure 7:**
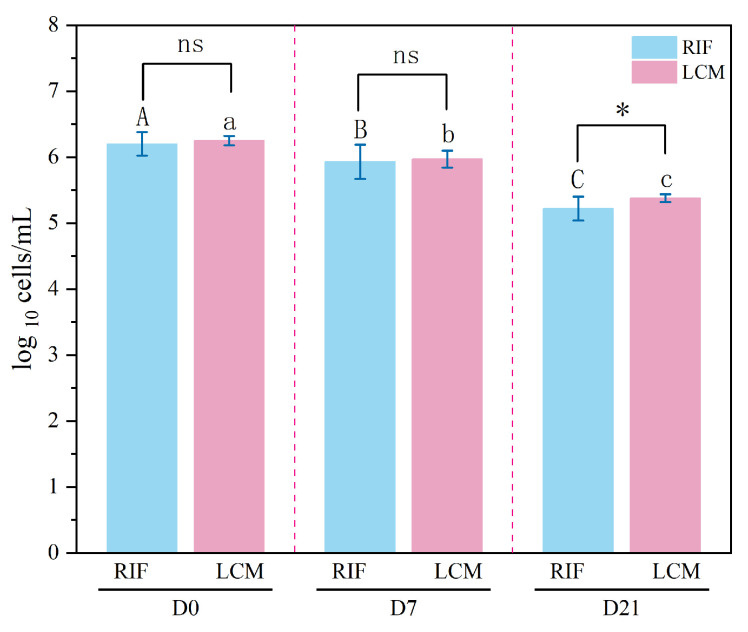
Results of milk somatic cells in rifaximin group and lincomycin group of cows (log_10_ cells/milliliter). Note: ∗ (*p* < 0.05) significantly different between the rifaximin group and lincomycin group; ns (*p* > 0.05) no significantly different between the rifaximin group and lincomycin group; the same capital letter means that there is no significant difference in rifaximin group (*p* > 0.05); and the same small letter means that there is no significant difference in Lincomycin group (*p* > 0.05).

Somatic cell counts are a critical indicator of mammary gland health, with elevated SCCs commonly associated with intramammary infections [54,55]. The Shapiro–Wilk test confirmed that SCCs did not follow a normal distribution at any time point (*p* < 0.05). Therefore, we performed a logarithmic transformation with a base of 10, as shown in Figure 7. Post hoc multiple comparisons aim to compare the SCC differences between the RIF treatment group and the LCM treatment group at each time point. These pairwise comparisons were conducted at each independent post treatment time point (i.e., D0, D7, and D21). Due to the significant difference in Mauchly sphericity test (*p* < 0.001), which violates the sphericity hypothesis, the Greenhouse Geisler correction is applied. Repeated ANOVA measures showed that the main effect of time was significant [F (1.42, 82.36) = 390.52, *p* < 0.001, η^2^ = 0.871], and the interaction between time and group was not significant [F (1.42, 82.36) = 1.804, *p* > 0.05, η^2^ = 0.030]. This indicates that the trend of somatic cell counts over time in the two groups of cows treated with rifaximin and lincomycin are parallel. In other words, the decline pattern of SCC did not differ depending on the antibiotics used. In D21, rifaximin (5.38 ± 0.06 log_10_ cells/mL) demonstrated a more pronounced reduction in SCCs compared to lincomycin (5.22 ± 0.18 log_10_ cells/mL) (*p* < 0.05), highlighting its potential as an effective therapeutic option for clinical mastitis. At the quarter level, the standard SCC for cattle from uninfected quarters often remains below 200,000 cells/mL [56], and the SCC of rifaximin group in our study fell below 200,000 cells/mL at D21. However, the more substantial decline in SCCs in the rifaximin group may reflect its superior bacteriological efficacy and anti-inflammatory properties. Previous studies have suggested that rifaximin’s localized action and minimal systemic absorption contribute to its potent antibacterial activity while also reducing the inflammatory response [18,30].

The correlation between SCC reduction and bacteriological cure, as stated by Degen et al. [57], was evident in our findings, where cows treated with rifaximin exhibited higher bacteriological cure rates and corresponding reductions in SCCs. Additionally, the SCC normalization in the rifaximin group at D7–D21 may suggest not only enhanced bacterial clearance but also a possible modulation of the local immune response. While these findings are promising, further study of the effects of rifaximin on subclinical mastitis is warranted to determine its broader applicability in mastitis treatment.

## 3. Materials and Methods

### 3.1. Reagents and Materials

Rifaximin intramammary infusion (lactating cow), with a specification of 100 mg of rifaximin per 5 mL tube, and lincomycin intramammary infusion (lactating cow), with a specification of 0.35 g of lincomycin per 5 g tube, were provided by Huaqinyuan Animal Pharmaceutical Co., Ltd. (Beijing, China). Rifaximin standard was bought from Sigma Chemical Company (St. Louis, MO, USA). Beijing Chemical Industry Co., Ltd. (Beijing, China) provided the liquid chromatography/mass spectrometry (LC/MS) grade formic acid. Acetonitrile (ACN) and methanol (MT) in LC/MS grade were procured from Fisher Scientific (Fair Lawn, NJ, USA). Anhydrous sodium sulfate (AR grade) was obtained from Sinopharm Chemical Reagent Co., Ltd. (Beijing, China). Solid-phase extraction (SPE) cartridge (Oasis HLB 500 mg 6 cc) and syringe-type microporous filter membrane (WAT202010, 0.22 µm) were purchased from Waters company (Milford, MA, USA).

### 3.2. Sample Preparation

1 g of milk sample was placed in a 15 mL centrifuge tube, adding 4 mL of ACN to precipitate the protein, and adding 1 g of anhydrous sodium sulfate (Na_2_SO_4_) to remove the water contained in the sample. After 1 min of swirling, the mixture was centrifuged for 8 min at 4300× *g*. The supernatant was placed in a clean 15 mL centrifuge tube, then the precipitate was mashed and extracted with 2 mL ACN. The supernatant was taken, and the extracts of the two extracts were combined. The extracts were separated and purified using an HLB cartridge that had been pre-activated with 5 mL MT and 5 mL water balance. At 40 °C, the eluent was dried by evaporation under a nitrogen stream. The residues were re-dissolved with 1 mL eluent A-eluent B (7/3, *v*/*v*) and filtered through a 0.22 µm syringe-type microporous filter membrane in advance of analysis.

### 3.3. UPLC-MS/MS Conditions

UPLC-MS/MS, which was equipped with a Xevo TQ-S mass detector (Waters, USA) and a Waters ACQUITY UPLC, was used to examine the extracted samples. Chromatographic separation was performed using a C_18_ reverse-phase column (ACQUITY UPLC^®^ BEH Phenyl column 1.7 µm, 50 mm × 2.1 mm; Waters, Milford, MA, USA). Column temperature was 40 °C. 0.1% formic acid in water (*v*/*v*) (eluent A) and acetonitrile (eluent B) served as the mobile phases for the gradient elution process. There was a 2 µL injection volume and a 0.3 mL/min flow rate. Table 5 displays the elution conditions for the gradient elution that was used. Multiple reaction monitoring (MRM) was used for MS analysis of rifaximin, and the Electrospray Ionization (ESI) source was in positive ion mode. Table 6 and Table 7 present the MS and MRM parameters, respectively.

### 3.4. Results of Method Validation

The analytical technique was validated in full compliance with EU regulations, Commission Decision 2002/657/EC [58]. To ensure adequate identification, confirmation and quantification of rifaximin in milk, the following method parameters were verified: selectivity, matrix effect, linearity, limit of detection (LOD), limit of quantification (LOQ), precision, accuracy, and stability.

#### 3.4.1. Selectivity and Matrix Effect

In this study, the chromatograms of blank mobile phase, 200 ng/mL rifaximin standard, blank milk matrix solution and blank milk matrix solution spiked with 200 ng/mL rifaximin were determined using UPLC-MS/MS. The specificity was ascertained by comparing the presence or absence of rifaximin peaks in these four chromatograms and other factors affecting the peak shape. Matrix effect was evaluated using the standard addition method by contrasting the peak areas of mobile phase and blank milk matrix spiked with 200 μg/kg of rifaximin. The relative matrix effect (ME) or matrix factor (MF) was calculated as:

$$\mathrm{ME}\;(\mathrm{standards})\;=\;\frac{\mathrm{peak}\mathrm{area}\mathrm{of}\mathrm{matrix}-\mathrm{matched}\mathrm{standards}}{\mathrm{peak}\mathrm{area}\mathrm{of}\mathrm{solution}\mathrm{standards}}$$

The coefficient of variation shall not be greater than 20% for the ME [58].

#### 3.4.2. Linearity

Matrix-matched standard solutions at a range of concentrations (1, 5, 20, 50, 100 and 200 ng/mL) were prepared by adding rifaximin standards to blank milk matrices. These six different concentrations of rifaximin solutions were assayed. The concentration of rifaximin was X and the peak area of rifaximin was Y. The weighted (1/x^2^) least square method was used to fit the standard curve.

#### 3.4.3. LOD and LOQ

The LOD and the LOQ were defined by detecting the addition of a given concentration of rifaximin to the blank milk matrices. The minimum level of concentration according to the signal-to-noise ratio (S/N) ≥ 3 was LOD, and the minimum level of concentration at a signal-to-noise ratio (S/N) ≥ 10 was LOQ.

#### 3.4.4. Precision and Accuracy

The accuracy and precision were verified by repeating the addition of a given concentration of rifaximin (1, 30, 60 and 120 ng/mL) to the blank milk matrices five times during a span of five consecutive days. The precision and accuracy were expressed as coefficient of variation (CV) and analyte recovery, respectively.

#### 3.4.5. Stability

The examination of the stability of rifaximin in milk samples includes short-term stability, long-term stability and repeated freeze–thaw stability. For 24 h, milk samples were kept at 25 °C to evaluate their short-term stability. Rifaximin was added to blank milk matrices prior to analysis and left for 30 days at −20 °C in order to study long-term stability. Samples were frozen at −20 °C and then were thawed at 25 °C through three such cycles to investigate the freeze–thaw stability.

### 3.5. Animal Experiment

In the current research, 96 Holstein lactating cows were included, and they were all provided by a dairy farm in Changping District, Beijing. The experimental unit was a single cow, and each cow was marked with an ear tag. Twelve healthy cows, weighing 611 ± 43 kg, aged 3–5 years, parity 2.81 ± 1.52, days in milk (DIM) 185.27 ± 43.81 days, were used for pharmacokinetic study. Twenty-four cows, weighing 670 ± 25 kg, aged 3 to 5 years, parity 3.15 ± 1.47, DIM 201.74 ± 29.08 days, were used for residue studies of rifaximin in cow milk. The remaining 60 cows used for pharmacodynamic study were with natural onset clinical mastitis, weighing 609.95 ± 29.69 kg, aged 3 to 6 years, parity 2.53 ± 1.67, DIM 150.27 ± 38.52 days, average milk yield of 30.15 ± 6.39 kg/day (7 days before onset of clinical mastitis). The cows utilized in the pharmacokinetic and residue studies were healthy cows. The cows utilized in the pharmacodynamic study had to meet the requirements that they only show clinical mastitis symptoms for one quarter. All the 96 cows had not received any antibiotic treatments in the past 30 days. The 96 cows were routinely fed concentrate-silage-haylage without any antibiotics, given water freely, and milked once in the morning and once in the evening throughout each experiment.

All animal experimental procedures were performed with due regard for animal welfare, according to the National Research Council (US) Guidelines for the Care and Use of Laboratory Animals [59], and ratified by the Committee on Animal Use and Care of the Feed Research Institute, Chinese Academy of Agricultural Sciences (No. FRI-CAAS-20190801).

#### 3.5.1. Milk Disposition Kinetics Study

According to the guiding principles for clinical pharmacokinetic tests of veterinary chemicals in Announcement No. 1247 of the Ministry of Agriculture of the People’s Republic of China, we selected 12 cows that met the specifications. According to directions, one rifaximin intramammary infusion was given in a single dose to one randomly selected gland of each cow after the first milk collection in the morning. Milk samples of 20 ± 5 mL were taken from each gland at 0 h before and 0.25, 0.75, 1, 4, 8, 12, 24, 48, and 72 h after administration. Prior to analysis, all milk samples were kept at −20 °C. The milk disposition kinetics for rifaximin in cows were analyzed with a non-compartment model, utilizing the Phoenix WinNonlin (version 8.1, Pharsight, Radnor, CA, USA).

#### 3.5.2. Residue Experiment

In this trial, ten cows produced more than 60 lb. (27.22 kg) of milk per day (early lactation) and ten cows less than 35 lb. (15.88) per day (late lactation), which met the regulatory requirements [60]. The administration of rifaximin was started in the night after the milking was completed, and one rifaximin intramammary infusion (5 mL: 100 mg) was injected into each quarter at 12 h intervals for three consecutive administrations. Blank milk samples were also collected from four untreated dairy cows as blank controls to establish the UPLC-MS/MS method for determining the residues of rifaximin in the milk samples collected. 20 ± 5 mL of mixed milk samples were taken from each quarter from each dairy cow at 12, 18, 24, 36, 42, 48, 60, 66, 72, 84, 90, 96 and 108 h after the last administration and were preserved at −20 °C before being analyzed. The withdrawal period of rifaximin for cow milk was calculated using WTM1.4 software.

#### 3.5.3. Efficacy Experiment

The criteria for conducting efficacy studies for intramammary products used in dairy cattle [43] were followed in this trial. The bacteriological cultures and clinical response are the main indications of a drug’s efficacy. In this study, the clinical response and bacteriological cultures’ results were used as the main indicators, and somatic cell count (SCC) was utilized as the reference indicator to comprehensively assess the efficacy of rifaximin as a treatment for natural onset of clinical mastitis in lactating cows, and the therapeutic effects were compared with the control drug lincomycin. Cows were randomly allocated to either the rifaximin treatment group or the control group by using SPSS (version 8.1) to generate random numbers and complete random design grouping. This ensured balanced distribution of potential confounders across groups. Double-blind design was applied in pharmacodynamics study. Treatment administrators were unaware of group assignments when administering interventions. Outcome assessors were blinded to treatment allocation to prevent measurement bias.

#### 3.5.4. Animals’ Treatment

Sixty cows, with one quarter exhibiting signs of clinical mastitis, were randomly allocated into two groups. Thirty cows in the experimental group were given rifaximin intramammary infusion at the recommended dose (one injection per quarter with mastitis) and thirty cows in the control group were given lincomycin intramammary infusion (lactating cow) at the recommended dose (one injection per quarter with mastitis). Rifaximin was administered 12 h apart for three consecutive injections. Lincomycin was administered 12 h apart for three consecutive doses. After administration, the clinical response was recorded, and milk samples were obtained for bacterial culture and somatic cell counts (SCCs).

#### 3.5.5. Assessment of Severity of Clinical Mastitis

At pre-treatment (Day 0, D0), and after treatment (D1, D2, D3, D4, D5, D6, D7, and D12), medical examinations including systemic observation, palpation of the mammary gland and visual observation of the milk were carried out on each cow, and clinical scores (CS) were assigned accordingly. The clinical score system referred to the previous description [61,62,63] and classified clinical cases of mastitis based on severity. Severity of clinical symptoms was scored as normal (CS = 0), mild (CS = 1), moderate (CS = 2), and severe (CS = 3) according to Table 8. The clinical scores for the appearance of milk and quarter were added together for the final score, and only cows with CS ≥ 2 were included in this trial. The SCC of the cows was examined with a somatic cell detector (Delaval, Switzerland) at D0, D7 and D21, respectively.

#### 3.5.6. Assessment of Bacteriological Cure

In accordance with the guideline [43], the following inclusion criteria were applied: For infectious mastitis caused by *S. aureus* or *Streptococcus*, animals were enrolled in the study if at least one pre-treatment milk sample yielded a positive culture. For mastitis caused by other bacterial pathogens, enrollment required two consecutive pre-treatment milk samples with positive culture results. One quarter was categorized as bacterial cure if there was no sign of bacterial growth in the milk sample after treatment. In addition to the original bacteria, the quarter in which other bacteria grow was also classified as the bacterial cure. Bacterial isolates (*E. coli*, *S. aureus*, *S. agalactiae*) were obtained from milk samples collected from a single commercial farm. Species identification was confirmed by 16S rRNA gene sequencing. The minimum inhibitory concentrations (MICs) of rifaximin against these isolates were determined in accordance with the Clinical and Laboratory Standards Institute (CLSI) guidelines, using the broth microdilution method [64]. *Staphylococcus aureus* ATCC 25923, *Escherichia coli* ATCC 25922, and *Streptococcus agalactiae* CVCC 598 were used as quality control strains. A detailed description of the microbiological methods and the complete MIC distributions are provided in the Supplementary Materials.

#### 3.5.7. Assessment of Somatic Cell Counts

The somatic cell counts of 60 cows were measured on the quarters diagnosed with mastitis using a somatic cell counter (Delaval, Tumba, Sweden) before administration (D0) and after administration (D7 and D21).

### 3.6. Statistical Analysis

The normality of clinical score data was assessed using the Shapiro–Wilk test. As the data were non-normally distributed, non-parametric methods were applied. Clinical scores are presented as median and interquartile range (IQR). Between-group comparisons (rifaximin vs. lincomycin) at each time point were performed using the Mann–Whitney U test, while within-group changes over time were analyzed with the Wilcoxon signed-rank test. A two-way repeated measures ANOVA was conducted to examine the effects of treatment group (RIF vs. LCM) and time (D0, D7, D21) on somatic cell count. Simple effects analysis was performed for significant interaction effects, and Tukey’s post hoc test was used for intergroup comparisons at each time point. The chi-square test was used to compare the clinical cure rates between the two groups at different time points. The overall mean and standard deviation (SD) were obtained from descriptive statistics in Tables S1 and S2, and the coefficient of variation within and between batches was calculated using one-way ANOVA analysis. All statistical analyses were conducted using IBM SPSS Statistics (version 26.0, IBM Corp., Armonk, NY, USA), the significance level is set at *p* < 0.05. Figure 4, Figure 6 and Figure 7 were generated using Origin (version 2021, OriginLab Corporation, Northampton, MA, USA).

## 4. Conclusions

The milk disposition kinetics and residue elimination of rifaximin intramammary infusion (lactating cows) in lactating cow milk were studied by establishing a UPLC-MS/MS method. Milk disposition kinetics parameters were obtained, and the milk withdrawal period was determined as 95.1 h. Additionally, the clinical cure rate and bacteriological cure rate of rifaximin intramammary infusion (lactating cows) in treating clinical mastitis were 10% higher than the control drug lincomycin intramammary infusion (lactating cows). At D21 of treatment with rifaximin, the SCCs was significantly lower than that of lincomycin (*p* < 0.05).

## Figures and Tables

**Table 1:** The main milk kinetics parameters of rifaximin (*n* = 12).

Milk Kinetics Parameters	Mean	SD
AUC (h⋅ng/mL)	340,731.8	43,968.82
T_1/2_ (h)	5.5748	0.68
λz (1/h)	0.1262	0.0166
MRT (h)	7.3927	1.3353
C_max_ (ng/mL)	54,273.33	12,421.32
AUMC (h⋅h⋅ng/mL)	2,475,745	230,305.1

Note: AUC (Area Under the Curve), the total exposure of the drug in milk over time, calculated as the area under the milk concentration-time curve; T_1/2_: (Elimination Half-Life), the time required for the drug concentration in milk to decrease by half during the terminal elimination phase; λz (Elimination Rate Constant), the first-order rate constant associated with the terminal (elimination) phase of the drug in milk; MRT (Mean Residence Time), the average total time that a single drug molecule resides in the milk within the mammary gland following administration; C_max_ (Maximum Concentration), the highest observed drug concentration measured in milk following administration; AUMC (Area Under the Moment Curve), the area under the first moment curve (concentration multiplied by time, versus time).

**Table 2:** The concentration of rifaximin in milk (*n* = 20).

Time (h)	Mean ± SD (μg/kg)
12	120,354 ± 39,209.74
18	68,770 ± 27,069.78
24	32,250 ± 13,478.23
36	14,756 ± 5858.79
42	6206 ± 2367.47
48	2373 ± 919.33
60	893.35 ± 217.35
66	409.05 ± 104.78
72	191.66 ± 63.82
84	68.1 ± 14.62
90	32.86 ± 9.90
96	14.86 ± 6.56
108	2.57 ± 2.10

**Table 3:** Clinical cure rates in cows with clinical mastitis in each group (*n* = 60, 30 cows per group).

Day	RIF Cure Rate	LCM Cure Rate	*p* Value	χ^2^
D4	20.00% (6/30)	13.33% (4/30)	0.490	0.476
D5	43.33% (13/30)	20.00% (6/30)	0.048	3.913
D6	66.67% (20/30)	43.33% (13/30)	0.048	3.333
D7	80.00% (24/30)	66.67% (20/30)	0.243	1.364
D12	83.33% (25/30)	73.33% (22/30)	0.359	0.842

**Table 4:** The detection rate of bacteria in each group (*n* = 60).

Pathogen	Group	Pre-Treatment		Post-Treatment	
		D − 1	D0	D14	D21
*Escherichia coli*	rifaximin	83.33%	83.33%	6.67%	6.67%
	lincomycin	90.00%	90.00%	16.67%	16.67%
*Staphylococcus aureus*	rifaximin	13.33%	13.33%	10.00%	10.00%
	lincomycin	16.67%	16.67%	16.67%	16.67%
*Streptococcus agalactiae*	rifaximin	10.00%	10.00%	6.67%	6.67%
	lincomycin	6.67%	6.67%	6.67%	6.67%

**Table 5:** The gradient elution conditions.

Run Time (min)	Eluent A (%)	Eluent B (%)
0.00	70.00	30.00
3.00	20.00	80.00
4.50	70.00	30.00

Note: eluent A, 0.1% formic acid in water (*v*/*v*); eluent B, acetonitrile.

**Table 6:** MS parameters.

Parameter	Settings
Ionization mode	Electrospray ionization (positive mode)
Capillary voltage	2.0 kV
Ion source temperature	150 °C
Desolvation temperature	400 °C
Cone gas flow	50 L/h
Desolvation gas flow	800 L/h
Secondary collision gas	Ar_2_

**Table 7:** MRM parameters.

Precursorion (*m*/*z*)	Production (*m*/*z*)	Cone Voltage (V)	Collision Energy (V)
786.6	150.8	30	50
	754.7 *	30	50

Note: * Quantitation ion.

**Table 8:** Clinical mastitis scoring system.

Observations	Appearance of Milk/Quarter	Clinical Score
**milk**	Normal	0
	Suspect (few transient flakes or clots) or small amounts of flocs	1
	Milk with large clots, flakes, or discoloration	2
	Milk with blood and/or pus, distinct odor	3
**quarter**	Normal	0
	Slight inflammation/swelling, warm to the touch, or both	1
	Moderate inflammation/swelling, hot to the touch, or both	2
	Severe inflammation/swelling, hot to the touch, or both	3

## Data Availability

All the gathered results are included in the present manuscript. Tables S1 and S2, please refer to the Supplementary Materials.

## Supplementary Materials

The following supporting information can be downloaded at: https://www.mdpi.com/article/10.3390/antibiotics14121203/s1, Table S1. The mean recovery, intra CV, and inter CV of rifaximin in cow milk (µg/kg). Table S2. Stability results of rifaximin in cow milk. Table S3. MIC distribution of rifaximin against a single-farm collection of cow mastitis isolates (*n* = 158).

## Abbreviations

The following abbreviations are used in this manuscript:

UPLC-MS/MS	Ultra performance liquid chromatography-tandem mass spectrometry
HPLC	High performance liquid chromatography
AUC	Area under curve
AUMC	Area under the moment curve
MRT	Mean residue time
Cmax	the peak concentration
Tmax	the peak time
LOD	Limit of detection
LOQ	Limit of quantifications
MRLs	Maximum residue limits
ME	Matrix effect
DIM	Days in milk
CV	Coefficient of variation
SCC	Somatic cell count
CS	Clinical scores
