# Supplementary material for: Milk Disposition Kinetics, Residue and Efficacy of Rifaximin After Intramammary Administration in Lactating Cow
Source: Antibiotics (Basel). 2025 Dec 1;14(12):1203. doi: 10.3390/antibiotics14121203 (PMC12730011; doi:10.3390/antibiotics14121203)
Supplement: Supplementary file 1 [file antibiotics-14-01203-s001.zip › antibiotics-3907085-supplementary.pdf]

Supplementary materials

Table S1. The mean recovery, intra CV, and inter CV of rifaximin in cow milk (μg/kg)

Spiked Concentration (μg/kg)	mean Recovery (%)	SD (%)	Intra CV (%) (n=5)					Inter CV (%) (n=25)
			1d	2d	3d	4d	5d	
**1**	77.65	3.05	7.42	12.78	2.45	7.26	7.09	8.20
**30**	80.15	5.21	2.32	7.93	4.74	2.02	4.39	7.28
**60**	82.94	2.58	5.53	9.21	4.74	1.00	1.15	5.59
**120**	85.98	1.97	1.15	6.84	4.83	0.29	7.71	5.14

Table S2. Stability results of rifaximin in cow milk

Spiked concentration（μg/kg）	Mean recovery (%) of rifaximin (Mean ± SD, n = 3)			
	0d	7d	30d	Repeated Freeze–Thaw
**1**	80.17± 2.08	77.07±3.56	75.65±2.90	79.16±4.52
**30**	81.17±3.93	80.69±3.52	79.81±3.36	78.47±3.51
**60**	82.58±3.01	80.62±4.96	83.55±4.25	79.85±5.03
**120**	85.48±3.52	85.28±2.40	83.42±2.08	85.26±2.31

Table S3. MIC distribution of rifaximin against a single-farm collection of cow mastitis isolates (n=158)

Micro-organism (no. of isolates)	MIC (μg/mL)		
	Range	MIC_{50}	MIC_{90}
Escherichia coli (*n* = 51)	2~8	4	8
Staphylococcus aureus (*n* = 57)	0.125	0.125	0.125
Streptococcus agalactiae (*n* = 50)	0.03125~16	0.5	8

Note: All isolates were obtained from a single commercial farm. The MIC distributions presented here should be interpreted as representative of the dominant clones circulating within this specific herd and do not reflect the genetic diversity of these pathogen species on a broader geographical scale

Supplementary Methods: Detailed Microbiological Procedures

Milk samples were collected from cow milk pre-treatment (D-1 and D0) and post-treatment (D14 and D21) for bacteriological cultures. Bacterial isolates were obtained on selective media including Mannitol Salt Agar, sheep blood agar, and MacConkey Agar. Representative colonies were selected for Gram staining and preliminary categorization. Based on the Gram reaction and colonial morphology, isolates were subjected to targeted

biochemical profiling. Gram-negative bacilli growing on MacConkey agar were tested using EMB, TSI, and IMViC assays, while Gram-positive cocci were differentiated using tests such as sodium hippurate hydrolysis and carbohydrate fermentation. All isolates were purified in brain heart infusion broth. Final identification was confirmed by sequencing the 16S rRNA gene using universal primers (27F/1492R) and comparing the sequences with the GenBank database by Biotechnology (Shanghai) Co., Ltd. An isolate was assigned to a species when its sequence showed ≥99% identity with the type strain sequence of that species in the database. Microbial susceptibility testing was conducted according to Clinical and Laboratory Standards Institute (CLSI) guidelines[64], employing the microbroth dilution method to determine the MIC of rifaximin against clinical isolates. Briefly, fresh bacterial colonies were suspended in saline to a turbidity equivalent to a 0.5 McFarland standard. This suspension was further diluted in Mueller-Hinton broth to achieve a final inoculum density of approximately 5×10^5 CFU/mL in each well of the custom-prepared microdilution panel. The rifaximin powder was dissolved in dimethyl sulfoxide (DMSO) and serially two-fold diluted across the panel, covering a concentration range of 0.06 to 64 μg/mL. The panels were incubated aerobically at 35°C for 18-20 hours. The MIC was defined as the lowest concentration of rifaximin that completely inhibited visible bacterial growth. *S. aureus* ATCC25923, *E. coli* ATCC 25922 and *S. agalactiae* CVCC598 were used as the quality control strains to ensure the accuracy of the test results. MIC_{50} and MIC_{90} values were calculated, representing concentrations inhibiting at least 50% and 90% of bacterial colony growth, respectively. The assay was performed in triplicate.
